# Comparison of various chemical compounds for the removal of SO_2_ and NO_x_ with wet scrubbing for marine diesel engines

**DOI:** 10.1007/s11356-021-16155-9

**Published:** 2021-09-08

**Authors:** Terence Chin, Ivan CK Tam, Chun-Yang Yin

**Affiliations:** 1 School of Applied Sciences, Nanyang Polytechnic, 569830 Singapore, Singapore; 2 Newcastle Research & Innovation Institute, Newcastle University, Singapore, 599493 Singapore

**Keywords:** NO, SO_2_, Wet scrubbing, Marine emissions, NaClO, NaClO_2_, KMnO_4_

## Abstract

Seawater, NaOH, NaClO, NaClO_2_, H_2_O_2_, and KMnO_4_ were used as scrubbing liquids to react with SO_x_ and NO_x_ separately in a customized wet scrubber. The absorption of SO_2_ in the aqueous phase was influenced by three factors: pH, ionic concentration, and oxidation potential. For NO_x_ removal, the effectiveness of various chemical compounds can be ranked from least to most effective as follows: seawater, NaOH, H_2_O_2_ < NaClO < KMnO_4_ < NaClO_2_. This effectiveness was influenced by the chemical compound’s ability to oxidize NO to NO_2_, absorb the NO_2_ that was formed, and retaining the nitrogen in the aqueous phase. High oxidation potential promoted the oxidation of NO to NO_2_ but hindered the absorption of NO_2_. NaClO_2_ was superior compared to NaClO in all three categories of oxidizing, absorption and retention. NaClO could not retain a significant amount of NO_2_ which it absorbed in the aqueous phase. The pH around 8 provided a good balance between oxidation versus absorption/retention and reactant utilization for the chlorine-based oxidants. KMnO_4_ had the lowest reactant consumption rate; only half a mole was consumed for every mole of NO removed, compared to around 2–3 mol of chlorite or 3–5 mol of hypochlorite.

## Introduction

The combustion of fossil fuels in power plants, boilers, and diesel engines are known to generate large amounts of air pollutants in the form of sulfur oxides (SO_x_) and nitrogen oxides (NO_x_), among other polluting substances. Control of these pollutants are particularly more challenging in the marine sector where ocean-plying ships with large diesel engines depend on low-cost heavy fuel oil for propulsion (Deng et al. 2021; Marine 2015). The application of known land-based end-of-pipe technologies onboard ocean-plying vessels faces many constraints, including space, weight, and logistical limitations.

The simplest solution for complying with the SO_x_ cap set in “Annex VI—Prevention of Air Pollution from Ships” of the International Convention for the Prevention of Pollution from Ships (MARPOL) would be to switch to low-sulfur fuel (Lloyd’s Register Marine 2015). However, this is estimated to cost the industry at least an additional several billion dollars per year due to the high cost of low sulfur fuel. Although once considered a fringe solution, the installation of wet scrubbers onboard vessels for SO_x_ removal is fast becoming mainstream (ABS Advisory On Exhaust Gas Scrubber Systems 2018).

Unfortunately, current commercial wet scrubbers are only effective for SO_x_ removal but not for NO_x_. The control of the latter has always been more challenging as most of it is generated in the engine itself due to the high temperatures of the combustion process (thermal NO_x_). Hence, it can be formed even if the fuel does not contain any nitrogen (Nevers 2000). Also, NO_x_ from engine combustion comprises around 90% of nitric oxide (NO), which is highly insoluble in the aqueous phase and cannot simply be scrubbed and solubilized from the gas to aqueous phase like SO_x_. Therefore, engine medication techniques are mainly used to reduce the combustion temperature in the engine so that less NO_x_ will be formed. However, such methods also reduce the engine efficiency, thereby leading to higher fuel consumption (Deng et al. 2021).

The acceptance of wet scrubbers onboard ships has already gained much traction—Wärtsilä itself has already built more than 700 units before 2019 (Ni et al. 2020). It is therefore justifiable to explore its potential to remove NO_x_ on top of SO_x_. Already in the R&D scene, there are many upcoming methods for treating SO_x_ and NO_x_ simultaneously. Out of these, a significant amount of work focused on gas–liquid reactions where strong oxidizing agents were used to oxidize NO to a more soluble NO_2_ form. The more common chemical compounds studied included chlorine-based oxidizing agents such as chlorite and hypochlorite (Brogren et al. 1998; Chien and Chu 2000; Chu et al. 2003; Deshwal and Lee 2009; Gong et al. 2020; Jin et al. 2006; Mondal and Chelluboyana 2013; Park et al. 2015; Wei et al. 2009; Yang et al. 2018; Zhao et al. 2010; Zhao et al. 2011; Zhao et al. 2015; Zhao et al. 2016), permanganates (Brogren et al. 1997; Chu et al. 2001; Fang et al. 2013), ozone (Han et al. 2020; Kang et al. 2020; Lin et al. 2016; Shao et al. 2019; Sun et al. 2017; Sun et al. 2015), and hydrogen peroxide (Liu et al. 2014; Liu and Zhang 2011; Wen et al. 2019; Wu et al. 2018; Xie et al. 2019).

Among these works, Yang et al. demonstrated that SO_2_ and NO can be effectively removed in a wet scrubbing system using hypochlorite-based oxidants that were directly generated by electrolysis of NaCl solution of similar concentration to seawater (Yang et al. 2018). Han et al. recently showed that continual dosing of chlorite oxidant in a pH buffered aqueous system could improve the reactant utilization rate (Han et al. 2019).

Although desulfurization using wet scrubbers in onboard vessels can already be considered established, there has been several interesting developments that has taken place recently. One of them involved the use of a cascading design in a wet scrubber that allowed for higher L/G ratio without the risk of flooding, thereby achieving higher sulfur removal, lower pressure drop, and with lower alkalinity requirements when compared with a straight-through traditional open-loop scrubber (Kuang et al. 2020; Zhao et al. 2021). In addition, a square-shaped spray column which allowed for a smaller footprint, smaller pressure drop, and higher efficiency was also proposed (Van Duc et al. 2021). Although only focused on desulfurization, these studies are interesting as some their innovations may be transferable to simultaneous SO_2_ and NO_x_ removal.

Although quite a few of these studies demonstrated full removal of SO_2_ and relatively high NO removal, many challenges remain; these include high reactant utilization rate, difficulty in removing NO_2_ that were generated from the oxidation of NO, potential release of toxic compounds in the exhaust (such as chlorine dioxide), and high nitrate content in the scrubbing wastewater. It is difficult to do an accurate comparison of these common wet scrubbing chemical compounds being studied because existing studies usually focuses on one of the substances that show potential but very little work is done on comparing multiple substances in a single experimental platform.

In the present study, a broad range of widely reported substances, namely, seawater, sodium hydroxide (NaOH), sodium hypochlorite (NaClO), sodium chlorite (NaClO_2_), hydrogen peroxide (H_2_O_2_), and potassium permanganate (KMnO_4_), were systematically compared for their capacity to remove SO_x_ and NO_x_ and new insights were gained from comparing these reactions together instead of separately. These chemicals were selected for this study because they either showed potential in NO_x_ removal, are widely available in the industry at a reasonable cost, or are already currently used on ship-based wet scrubbers.

Besides a wide range of reactants being compared, other novelties in the work here include the comparison of the consumption rate and chemical cost among various reactants, and observations of the influence of oxidation reduction potential (ORP) values in the various reaction mechanisms, which are seldom reported in existing studies. Other important reaction characteristics such as removal efficiency, change in pH, change of ionic concentration in the aqueous phase before and after reaction, and reactant stability were compared and discussed here. Existing challenges faced in current research, especially the difficulty in absorbing the NO_2_ that has been formed after the oxidation of NO, will be discussed thoroughly based on the comparison between the various chemical reactions from the wide range of compounds studied.

## Materials and methods

### Experimental system

The sodium hydroxide (NaOH) solution used was from Merck Millipore (Titripur series), 1 M concentration. Both sodium chlorite (NaClO_2_) (80% assay) and potassium permanganate (98% assay) were from Acros Organics. Hydrogen peroxide (H_2_O_2_) was purchased from VWR Chemicals, with a concentration of 6% w/v in solution form. The sodium hypochlorite (NaClO) reagent grade solution used was purchased from Sigma-Aldrich, with available chlorine of 4.00–4.99%. Its actual concentration was determined by iodometric titration according to ASTM 15.04 D 2022. Seawater used for experiments were collected from the Labrador Park jetty, Singapore.

**Fig. 1:**
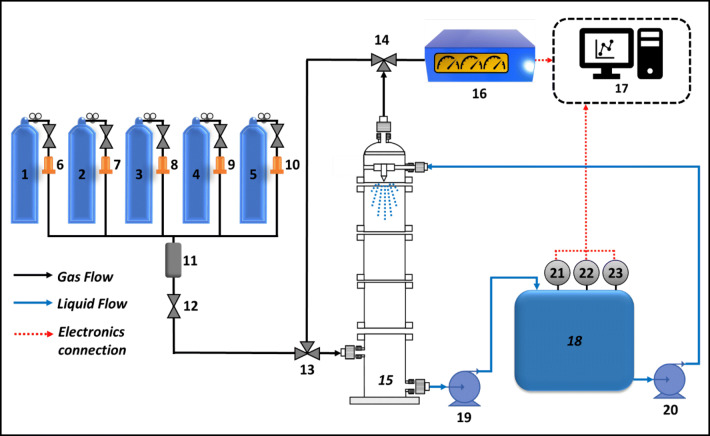
Schematic diagram representing the experimental setup: (1–5) gas cylinders, N_2_, O_2_, SO_2_, NO, and NO_2_; (6–10) mass flow controllers; (11) gas mixer; (12) ball valve; (13–14) three-way valves; (15) adjustable glass scrubber; (16) flue gas analyzers; (17) datalogging computer; (18) scrubbing liquid tank; (19–20) peristaltic pumps; (21) pH meter; (22) conductivity meter; (23) ORP meter

The sulfur dioxide (SO_2_) and nitric oxide (NO) gases used in this experiment were blended at a concentration of 10,000 ppm(v) in nitrogen. These, together with the oxygen (99.99%) and nitrogen (99.995%) gases, were supplied by Singapore Oxygen Air Liquide Private Limited. The nitrogen dioxide (NO_2_) gas at a concentration of 5000 ppm(v) was supplied by Leedon National Oxygen Ltd. Various gases from the gas cylinders were mixed in the appropriate ratio and are hence referred to as “simulated exhaust gas” (Fig. 1).

Two different flue gas analyzers were used in this setup. The first was the Testo 350XL that used electrochemical cell sensors to measure O_2_, SO_2_, and NO. For NO_2_ gas, it was observed that the values measured by the electrochemical cell were affected by the presence of chlorine-based gases. Therefore, a second analyzer was used, which was the MGA Luxx containing an NDIR-based NO_2_ sensor. The NDIR-based NO_2_ sensor was more accurate as it was not subjected to cross-referencing interferences experienced by electrochemical sensors. Moisture from the gas was removed by in-built moisture traps in both analyzers. Data from both the exhaust gas analyzers were data-logged with their respective software and the NO_x_ data was calculated by using NO_x_ = NO + NO_2_.

The wet scrubber used is a custom-made glass wet scrubber with adjustable height and an internal diameter of around 100 mm. A filter disc was built at the top of the scrubber to function as a mist eliminator to retain the liquid in the scrubber. The pH, conductivity, and ORP probes used to measure and data-log the scrubbing liquid in the tank were from Thermo Fisher Scientific.

The Basic 792 Ion Chromatograph system by Metrohm was used for quantitative analysis of ${\mathrm{NO}}_2^{-}$, ${\mathrm{NO}}_3^{-}$, ${\mathrm{Cl}}_2^{-}$, and ${\mathrm{ClO}}_2^{-}$ while the Libra S22 UV-VIS Spectrophotometer by Biochrome was used for ClO^−^ and ${\mathrm{MnO}}_4^{-}$. ${\mathrm{MnO}}_4^{-}$ aqueous samples were analyzed with the UV-Vis only and not the ion chromatograph in order to protect the instrument and avoid any staining or damaging of the column.

### Experimental procedure

A three-way valve was used to manually switch the simulated exhaust gas directly to the flue gas analyzers before reaction and from the exit of the wet scrubber during reaction. The experimental conditions used in this study are found in Table 1.

**Table 1:** Experimental conditions used

**Simulated exhaust gas properties**	
Composition (for SO_2_ study)	SO_2_: 500 ppm(v)/O_2_: 14%/N_2_ balance
Composition (for NO study)	NO: 500 ppm(v)/O_2_: 14%/N_2_
Gas flowrate	10 L/min
Temperature	Ambient (~25 °C)
**Scrubbing liquid properties**	
Volume used	2.5 L
Liquid flowrate	1.0 L/min
**Wet scrubber parameters**	
Spray nozzle type	Promax Quick Fulljet QPHA-3 (capacity 1.1 L/min)
Active height of wet scrubber	430 mm
Diameter	99 mm
Packing material	Nil
Space velocity	0.022 m/s
Residence time	19.9 s
**Others**	
Experiment duration	60 min

Two separate reactions were planned for the scrubbing liquid mixtures being studied—the first for SO_2_ removal and the second for NO removal. In each experiment, 2.5 L of the scrubbing solution were recirculated through the wet scrubber to react with the simulated exhaust gas for 60 min. A third study involving the removal of NO_2_ was added for selected scrubbing mixtures to obtain better clarity on the subject. A lower L/G ratio was also studied for reaction with NO, where the L/G ratio was reduced to 15 L/m^3^. Here, the gas flowrate was increased to 25 L/min, the spray nozzle used was the QPHA-1 (0.38 L/min) and the experiment time reduced to 30 min. The rest of the parameters remained as stated in Table 1.

The properties of the different types of chemicals used as scrubbing liquids for the gas–liquid reaction can be found in Table 2. Among the oxidizing agents used in the scrubbing liquid, it is known that the oxidation potential of both NaClO and NaClO_2_ are highly pH sensitive. Therefore, both the scrubbing liquids of these compounds were also adjusted to pH 6 and 8 before the start of the reaction using 0.1 M of hydrochloric acid. When prepared without any pH adjustments, the starting pH of NaClO and NaClO_2_ were 10.9 and 10.6, so these liquid mixtures were designated as NaClO/pH10.9 and NaClO_2_/pH10.6 respectively.

**Table 2:** Composition and properties of the scrubbing liquid used to react with the exhaust gas in the wet scrubber for the SO_2_ and NO studies, respectively

No	Scrubbing liquid composition	Concentration (M)	Total Alkalinity (mg/L CaCO_3_)	Starting measurement			SO_2_ study	NO study	NO_2_ study
				pH	ORP (mV)	Cond. (mS/cm)			
1	Seawater (SW)	NA	116	8.2	206	47.2	x	x	–
2	NaOH	0.05	2468	12.4	−5	11.2	x	x	x
		0.10	–	12.9	−15	21.1	–	–	x
		0.40	–	13.4	−32	75.7	–	–	x
3	H_2_O_2_	0.05	6	4.4	352	–	x	x	–
4	NaClO/pH10.9	0.05	436	10.9	625	10.4	x	x	–
5	NaClO/pH 8	0.05	–	7.9	880	10.5	–	x	–
6	NaClO/pH 6	0.05	–	6.0	1013	11.4	–	x	–
7	NaClO_2_/pH10.6	0.05	350	10.6	432	6.0	x	x	x
8	NaClO_2_/pH 8	0.05	–	8.3	483	5.8	–	x	x
9	NaClO_2_/pH7	0.05	–	7.3	–	5.8	–	–	x
10	NaClO_2_/pH 6	0.05	–	6.2	523	5.8	–	x	–
11	KMnO_4_	0.05	36	9.2	561	6.0	x	x	–
12	Deionized (DI) water	NA	4	6.2	353	0.006	x	–	x

For reactions that were able to remove NO_x_, aqueous samples were collected at the beginning, midpoint, and the end of the experiments (0, 30, and 60 min, respectively) for ionic analysis within 24 h of the experimental run.

### Calculations

The removal efficiency (*ƞ*_*i*_) of SO_2_, NO, NO_2_, and NO_x_ by the various gas–liquid reactions were calculated according to Eq. (1):

1 $$ {\eta}_i=\frac{C_{i, inlet}-{C}_{i, outlet}}{C_{i, inlet}}\times 100\% $$

where *C*_*i*, *inlet*_ and *C*_*i*, *outlet*_ refer to the concentrations of the gas pollutants at the inlets and outlets of the wet scrubber, as recorded by the flue gas analyzers. For Figs. 9 and 10, the values of *NO*_*2*_ *formed* and *NO*_*2*_ *absorbed* were estimated from the flue gas analyzer data—it was assumed that NO removed was equivalent to *NO*_*2*_ *formed* and *NO*_*2*_ *absorbed* was equivalent to NO_x_ removed.

The amount of NO_x_ removed (in mmol) by various scrubbing mixtures shown in Table 5 was calculated by taking the area under the curve in the graphs of NO_x_ removal versus time. The NO_x_ removed (in ppm(v)) was converted to moles using the ideal gas law (*PV = nRT*), at 1 atm and 25 °C.

## Results and discussion

### Removal of sulfur oxides (SO_x_)

The reaction mechanisms for the absorption of SO_2_ in the aqueous phase have been well documented and can be summarized as follows (Al-Enezi et al. 2001; Andreasen and Mayer 2007; Tokumura et al. 2006):

2 $$ \mathrm{S}{\mathrm{O}}_2\left(\mathrm{g}\right)\to \mathrm{S}{\mathrm{O}}_2\left(\mathrm{aq}\right) $$

3 $$ \mathrm{S}{\mathrm{O}}_2\left(\mathrm{g}\right)+{\mathrm{H}}_2\mathrm{O}\to \mathrm{HS}{\mathrm{O}}_3^{-}+{\mathrm{H}}^{+} $$

4 $$ \mathrm{HS}{\mathrm{O}}_3^{-}\to \mathrm{S}{\mathrm{O}}_3^{-}+{\mathrm{H}}^{+} $$

5 $$ \mathrm{HS}{\mathrm{O}}_3^{-}+\frac{1}{2}{O}_2\left(\mathrm{g}\right)\to \mathrm{S}{\mathrm{O}}_4^{2-}+{\mathrm{H}}^{+} $$

6 $$ \mathrm{S}{\mathrm{O}}_3^{-}+\frac{1}{2}{O}_2\left(\mathrm{g}\right)\to \mathrm{S}{\mathrm{O}}_4^{2-} $$

These series of equations can roughly be grouped into two categories, the first being the absorption of SO_2_ into bisulfite or sulfite in the aqueous phase (Eqs. 3 and 4), followed by oxidation to sulfate, its most stable aqueous form (Eqs. 5 and 6). From the reactions, it can be seen that the absorption of 1 mol of SO_2_ in the aqueous phase results in the release of 1 to 2 mol of protons, thereby causing the pH to reduce over time.

**Fig. 2:**
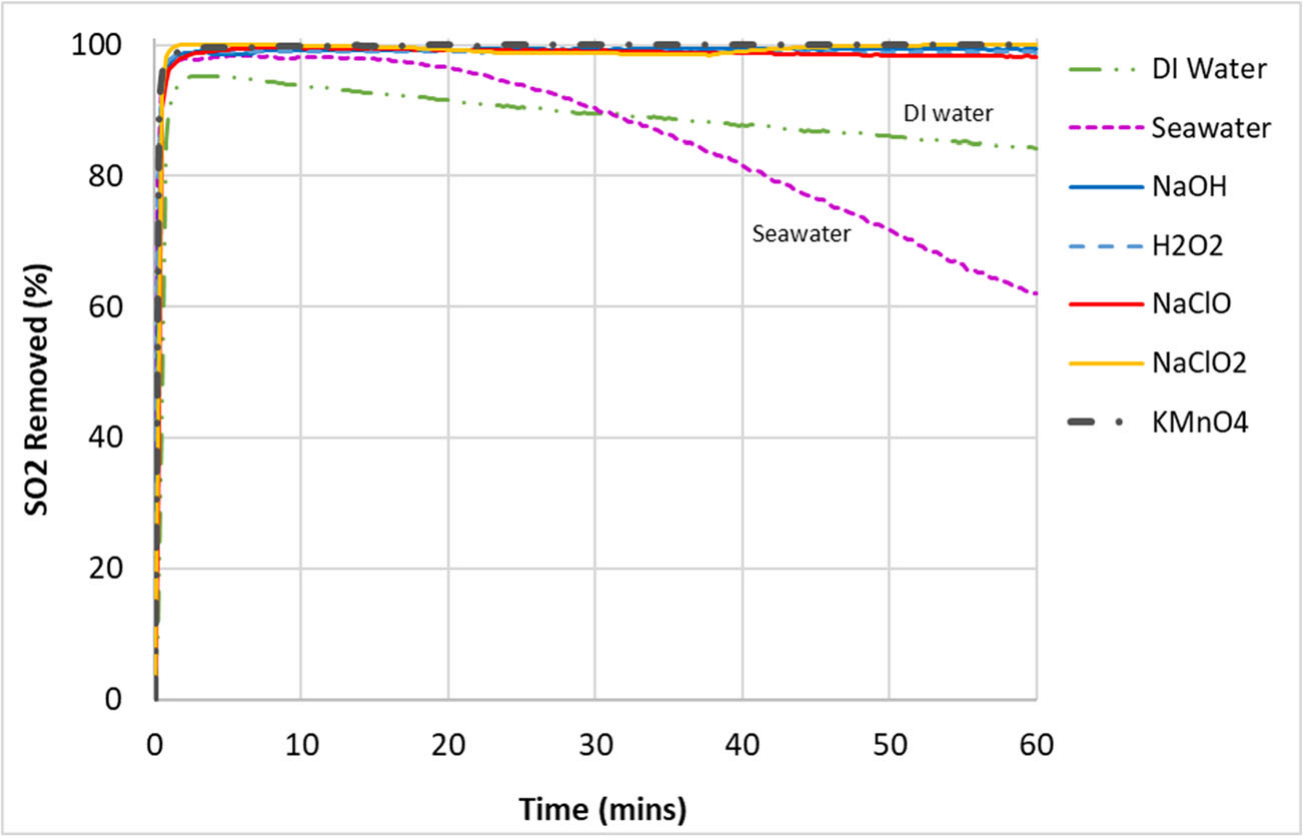
Sulfur dioxide (SO_2_) removal from the simulated exhaust gas with various scrubbing liquids

**Fig. 3:**
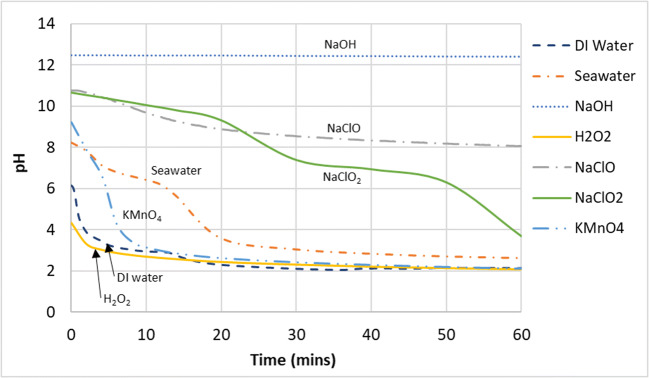
pH of the scrubbing liquid in the tank during SO_2_ removal

**Fig. 4:**
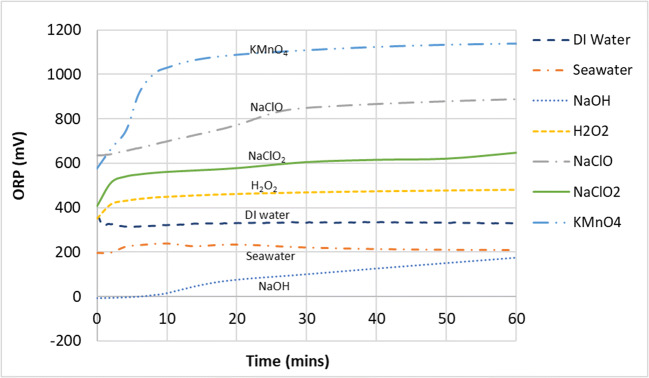
ORP of the scrubbing liquid in the tank during SO_2_ removal

The removal of SO_2_ by the gas–liquid reaction in the wet scrubber by various scrubbing mixtures can be seen in Fig. 2 while the corresponding pH and ORP values are shown in Figs. 3 and 4, respectively. It can be seen from Fig. 2 that all compounds used achieved 100% SO_2_ removal for the entire duration of the experiments except for DI water and seawater.

The reduction in SO_2_ removed seen in DI water and seawater can be explained by the gradual reduction of pH over time as the reaction progressed. Further comparison between DI water and seawater showed that the performance of the seawater in removing SO_2_ dipped below that of DI water at about the halfway point of the experiment although its pH still remained higher than the pH of DI water. This showed that other mechanisms could be at play for SO_2_ removal besides pH.

There are two possibilities for this. First, DI water was more oxidative compared to seawater (Fig. 4), which could have aided the oxidation of sulfite and bisulfite ions into sulfate ions (Eqs. 5 and 6) and helped in the overall removal of SO_2_. Second, seawater is much more saturated with ions compared to DI water, and this can extensively lessen the solubility of gases in the aqueous phase (﻿Black & Veatch Corporation, 2009). This vast difference in ionic saturation can be seen it their conductivity values—the conductivity of seawater at 47.2 mS/cm was more than 7400 times higher than the conductivity of DI water (Table 2). Furthermore, as typical seawater already contains around 2500–3000 mg/L of sulfate ions (Al-Enezi et al. 2001; Andreasen and Mayer 2007; Vidal and Ollero 2001; Black & Veatch Corporation, 2009), the saturation level of sulfur ions in the aqueous phase will be reached more quickly due to the common ion effect compared with DI water, which was almost free of ions.

Although the scrubbing mixtures of KMnO_4_ and H_2_O_2_ were on par or even more acidic than DI water and seawater in terms of pH, they both performed better in terms of SO_2_ removal. Again, this could be due to the higher oxidation potential of KMnO_4_ and H_2_O_2_, giving them an advantage in the oxidation of sulfite and bisulfite ions into sulfates. It can be summarized that the full removal of SO_2_ proceeded quite readily and was achieved by nearly all types of scrubbing mixtures that were tested here. This is because SO_2_ gas is very soluble in the aqueous phase (Table 3) (Sander 2015). It was observed that the absorption of SO_2_ in the aqueous phase under the experimental conditions here were likely influenced by three factors, namely, pH/alkalinity, concentration of soluble sulfur ions already present in the solution, and oxidation potential (estimated by ORP). An effective scrubbing liquid for the removal of SO_2_ gas should have a high pH or alkalinity, low in sulfate ions, and oxidative in nature. If equipment for the measurement and monitoring of sulfite or sulfate ions in the aqueous phase is not available, a conductivity meter could be used to determine the overall ionic content as a substitute of sorts to provide some indication.

**Table 3:** Henry’s law constant for several gases of interest, at 1 atm and 25 °C (Sander 2015)

Gas	Henry’s law constant, H_CO2_ (mol.m^−3^/Pa)	Solubility compared to NO
Carbon dioxide, CO_2_	3.3 ×10^−4^	17.4
Sulfur dioxide, SO_2_	1.3 ×10^−2^	684
Nitric oxide, NO	1.9 ×10^−5^	1
Nitrogen dioxide, NO_2_	9.9 ×10^−5^	5.2

### Removal of nitrogen oxides (NO_x_)

Under natural conditions, the removal of NO when it is released into the atmosphere is a two-part mechanism, according to the following reactions (Nevers 2000; Yang et al. 2018):

7 $$ \mathrm{NO}\left(\mathrm{g}\right)+\frac{1}{2}{\mathrm{O}}_2\left(\mathrm{g}\right)\to {\mathrm{NO}}_2\left(\mathrm{g}\right)\kern1em $$

8 $$ 3{\mathrm{NO}}_2\left(\mathrm{g}\right)+{\mathrm{H}}_2\mathrm{O}\left(\mathrm{g}\right)\leftrightarrow 2\mathrm{H}{\mathrm{NO}}_3\left(\mathrm{aq}\right)+\mathrm{NO}\left(\mathrm{g}\right) $$

9 $$ 2{\mathrm{NO}}_2\left(\mathrm{g}\right)+{\mathrm{H}}_2\mathrm{O}\left(\mathrm{g}\right)\leftrightarrow \mathrm{H}{\mathrm{NO}}_2\left(\mathrm{aq}\right)+\mathrm{HN}{\mathrm{O}}_3\left(\mathrm{g}\right) $$

In the first step, NO is naturally oxidized into a more soluble form, NO_2_ (Eq. 7), before it is subsequently solubilized (Eqs. 8 and 9). Although the oxidation of NO to NO_2_ takes place spontaneously under atmospheric conditions, the rate of reaction is relatively slow and there is insufficient time for this to occur in the combustion process before the exhaust is discharged. Therefore, a substantial amount of research has focused on speeding up this oxidation process so that the absorption step in the aqueous phase can take place.

In addition to the reactions shown in Eqs. 8 and 9 for the absorption of NO_2_, Brogren and Deshwal suggested additional pathways involving the formation of more soluble intermediate in N_2_O_3_ and N_2_O_4_ (Brogren et al. 1998; Deshwal et al. 2008):

10 $$ \mathrm{NO}\left(\mathrm{g}\right)+\mathrm{N}{\mathrm{O}}_2\left(\mathrm{g}\right)\leftrightarrow {\mathrm{N}}_2{\mathrm{O}}_3\left(\mathrm{g}\right)\kern0.75em $$

11 $$ 2\mathrm{N}{\mathrm{O}}_2\left(\mathrm{g}\right)\leftrightarrow {\mathrm{N}}_2{\mathrm{O}}_4\left(\mathrm{g}\right)\kern0.75em $$

12 $$ {\mathrm{N}}_2{\mathrm{O}}_3\left(\mathrm{g}\right)+{\mathrm{H}}_2\mathrm{O}\leftrightarrow \mathrm{HN}{\mathrm{O}}_2\left(\mathrm{aq}\right)\kern1.25em $$

13 $$ {\mathrm{N}}_2{\mathrm{O}}_4\left(\mathrm{g}\right)+{\mathrm{H}}_2\mathrm{O}\leftrightarrow 2{H}^{+}+\mathrm{N}{O}_2^{-}+\mathrm{N}{O}_3^{-}\kern0.50em $$

It was further demonstrated by Sun, Lin, and Shao that the formation of intermediate N_2_O_5_ was also very effective for the solubilization of nitrogen in the aqueous phase (Lin et al. 2016; Shao et al. 2019; Sun et al. 2015). It is generally accepted that the solubility of gaseous nitrogen in the aqueous phase increases with increasing nitrogen valency (Lin et al. 2016).

### Oxidation of nitric oxide (NO)

**Fig. 5:**
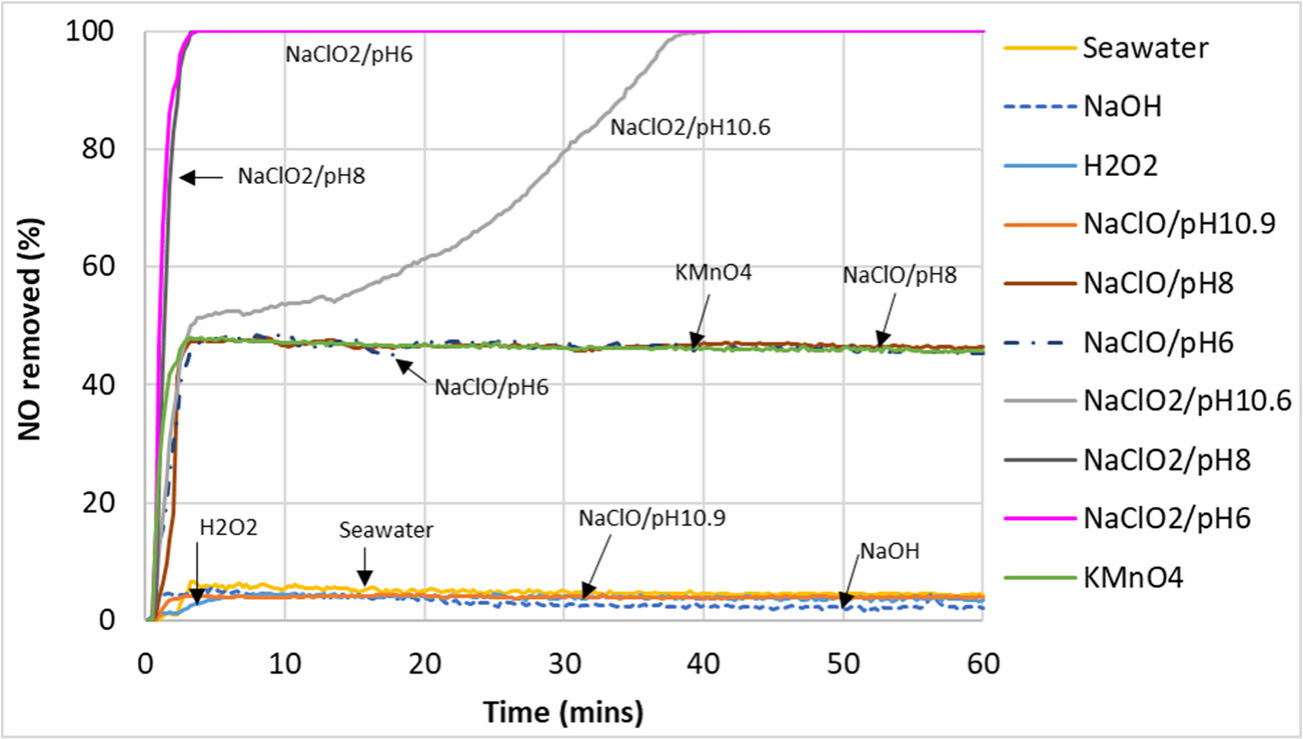
Nitric oxide (NO) removal from the simulated exhaust gas with various scrubbing liquids

The results of the removal of nitric oxide (NO) from the simulated exhaust gas from the gas–liquid reaction in the wet scrubber can be seen in Fig. 5. Since the removed NO could have been oxidized to NO_2_ without the latter getting absorbed, it should be noted that NO removal does not necessarily translate to NO_x_ removal.

The outcome can be broadly categorized into three groups—ineffective, somewhat effective, and effective. In the ineffective group, it can be seen that seawater, H_2_O_2_, NaOH, and NaClO/pH10.9 converted less than 5% of nitric oxide in the flue gas throughout the entire duration of the experiment. That is why existing commercial marine wet scrubbers which use seawater and NaOH are able to remove SO_2_ effectively but not NO.

In the somewhat effective group, KMnO_4_, NaClO/pH6, and NaClO/pH8 removed around 50% of the nitric oxide in the simulated emission gas. In the effective group, NaClO_2_/pH6 and NaClO_2_/pH8 achieved 100% nitric oxide removal for the duration of the experiment. The NaClO_2_/pH10.6 aqueous mixture could only remove around 50% of nitric at the beginning, but its removal efficiency continued to increase as the reaction progressed until it eventually reached 100% removal.

**Fig. 6:**
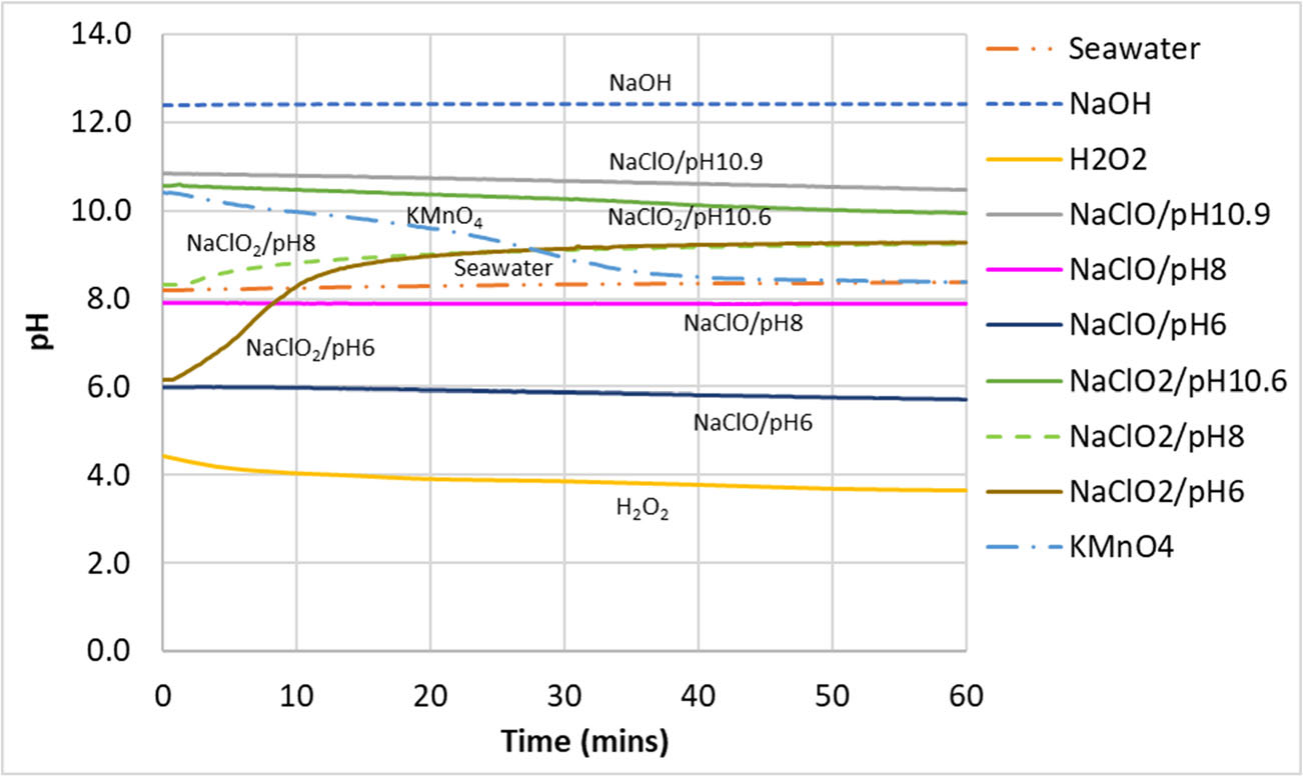
pH of the scrubbing liquid in the tank during NO removal

**Fig. 7:**
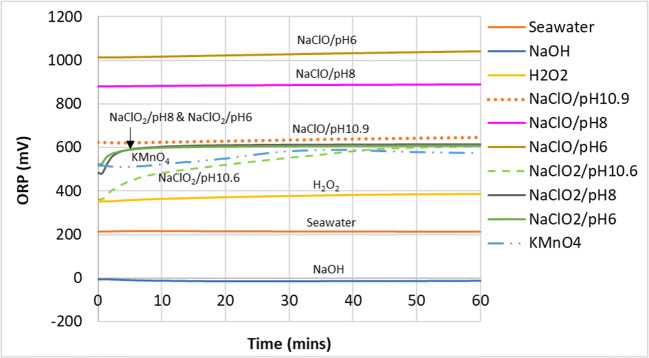
ORP of the scrubbing liquid in the tank during NO removal

As seen in the pH graph in Fig. 6, high pH (NaOH) has no effect on the removal of NO gas as this gas–liquid reaction does not seem to follow an acid–alkaline absorption reaction owing to its very low solubility (Table 3). Observation of the ORP change (Fig. 7) showed that NaClO mixtures had higher values than NaClO_2_ and KMnO_4_ but performed worse in the conversion of NO. This showed that solely using ORP values to predict the effectiveness of a liquid mixture to oxidize NO to NO_2_ would be inaccurate. However, ORP values did give a good indication of NO conversion when predicting the effectiveness of the same chemical compounds under different mixing conditions (pH, concentration, etc).

### Oxidation of nitric oxide by hydrogen peroxide (H_2_O_2_)

H_2_O_2_ was not effective in oxidizing NO to NO_2_ in this study. On top of using a high concentration, studies involving H_2_O_2_ usually required some sort of activation either with UV radiation or ozone (Wen et al. 2019; Xie et al. 2019). Without these, it can be seen that H_2_O_2_ was inferior compared to other types of chemical oxidants of the same concentration studied here.

### Oxidation of nitric oxide by sodium hypochlorite (NaClO)

NaClO was ineffective in removing NO gas in the wet scrubber in high pH but was more effective when the starting pH was lowered to 8 and 6, respectively. It is widely known that hypochlorites will partition itself between its ionic and hypochlorous acid form according to pH. It exists as hypochlorous acid (HOCl) below pH 6, as hypochlorite (ClO^−^) above pH 10, and a mixture of two between pH 6 and 9 (Metcalf et al. 1991).

When NaClO was at pH 10.9, the chlorine existed in its hypochlorite form (ClO^−^) where it is a less powerful oxidizing agent. As the pH was decreased to 8, partitioning into the hychlorous acid form began and the oxidation potential increased as a result (Fig. 7). This trend was also consistent with theoretical values shown in Table 4—the half reaction of the oxidation of hypochlorous acid was +1.49 V, much higher compared to its hypochlorite at +0.90 V (Tchobanoglous et al. 2013). However, futher lowering of pH from 8 to 6 did not show additional improvement in removal effectiveness, which was consistent with the findings of Yang et al. (2016).

**Table 4:** The oxidation potential of various chemical compounds of relevance in this study (Tchobanoglous et al. 2013)

Chemical compounds	Half reaction	Oxidation potential, V
Hydrogen peroxide	H_2_O_2_ + 2H^+^ + 2e^−^ ↔ 2H_2_O	+1.78
Permanganate	$\mathrm{Mn}{\mathrm{O}}_4^{-}+4{\mathrm{H}}^{+}+3{\mathrm{e}}^{-}\leftrightarrow \mathrm{Mn}{\mathrm{O}}_2+2{\mathrm{H}}_2\mathrm{O}$	+1.67
Chlorine dioxide	${\mathrm{ClO}}_2+{\mathrm{e}}^{-}\leftrightarrow {\mathrm{ClO}}_2^{-}$	+1.50
Hypochlorous acid	HOCl + H^+^ + 2e^−^ ↔ Cl^−^ + H_2_O	+1.49
Hypochlorite	ClO^−^ + H_2_O + 2e^−^ ↔ Cl^−^ + 2OH^−^	+0.90
Chlorite	$\mathrm{Cl}{O}_2^{-}+{\mathrm{H}}_2\mathrm{O}+4{\mathrm{e}}^{-}\leftrightarrow {\mathrm{Cl}}^{-}+4\mathrm{O}{\mathrm{H}}^{-}$	+0.76

The reaction mechanism of nitric oxide conversion by sodium chlorite is likely via the following pathways (Mondal and Chelluboyana 2013; Yang et al. 2016; Yang et al. 2018):

14 $$ \mathrm{N}\mathrm{O}\left(\mathrm{g}\right)+\mathrm{HOCl}\to \mathrm{N}{\mathrm{O}}_2\left(\mathrm{aq}\right)+{\mathrm{H}}^{+}+\mathrm{C}{\mathrm{l}}^{-} $$

15 $$ \mathrm{N}\mathrm{O}\left(\mathrm{g}\right)+{\mathrm{ClO}}^{-}\to \mathrm{N}{\mathrm{O}}_2\left(\mathrm{aq}\right)+\mathrm{C}{\mathrm{l}}^{-} $$

### Oxidation of nitric oxide by sodium chlorite (NaClO_2_)

Chlorite’s lower oxidation potential seen in the ORP readings (Fig. 7) was consistent with theoretical values seen in Table 4 (+0.76 vs. +0.90 to 1.49 V of the hypochlorite/hypochlorous combination). Yet, it outperformed hypochlorite in oxidizing NO to NO_2_. This was likely due to the tendency of NaClO_2_ to decompose into its more oxidative form, ClO_2_, at more acidic pHs, according to the reactions shown in Eqs. 16 and 17 (Choudhury 2011; Gong et al. 2020; Zhao et al. 2015). This decomposition will occur slowly when the pH is from 5 to 7 and accelerate when the pH is below 5.

16 $$ 5\mathrm{Cl}{\mathrm{O}}_2^{-}\left(\mathrm{aq}\right)+4{\mathrm{H}}^{+}\to 4\mathrm{Cl}{\mathrm{O}}_2\left(\mathrm{aq}\right)+\mathrm{C}{\mathrm{l}}^{-}\left(\mathrm{aq}\right)+2{\mathrm{H}}_2\mathrm{O} $$

17 $$ \mathrm{Cl}{\mathrm{O}}_2\left(\mathrm{aq}\right)\to \mathrm{Cl}{\mathrm{O}}_2\left(\mathrm{g}\right) $$

Further evidence of this decomposition can be seen from Fig. 6 where the pH for the scrubbing mixtures of NaClO_2_/pH6 and NaClO_2_/pH8 defied the common trend by increasing instead of decreasing as the reaction progressed even though the reaction with NO_x_ generates proton ions. This was likely because the conversion of chlorite to chlorine dioxide absorbs proton ions, according to Eq. 16.

When the pH was in the alkaline range, it was likely that any decomposition from $\mathrm{Cl}{\mathrm{O}}_2^{-}$ into ClO_2_ gas was likely to have occurred mainly at the gas–liquid boundary layer when the chlorite ion came into contact with the NO gas instead of the bulk phase (Gong et al. 2020). This mechanism enabled the chlorite solution to effectively oxidize NO to NO_2_ without needing very high ORP values in the bulk solution.

It could be seen from Figs. 6 and 7 that in general, the ORP values increase with decreasing pH, due to the increasing decomposition of $\mathrm{Cl}{\mathrm{O}}_2^{-}$ to ClO_2_. However, it can be seen that in the pH range of 6 to 8, the ORP values were quite similar, hovering at around 600 mV. This suggested that further pH adjustment below 8 seemed unnecessary and does not increase the oxidation potential of the solution. Higher than necessary formation of the more volatile ClO_2_ would result in more reactant being lost to the exhaust, which is both wasteful and a potential safety hazard. The reaction mechanism for the conversion of NO to NO_2_ by sodium chlorite is likely to have taken place in the following manner (Deshwal et al. 2008):

18 $$ 2\mathrm{N}\mathrm{O}\left(\mathrm{g}\right)+{\mathrm{Cl}\mathrm{O}}_2^{-}\ \left(\mathrm{aq}\right)\to 2\mathrm{N}{\mathrm{O}}_2\left(\mathrm{g}\right)+{\mathrm{Cl}}^{-}\left(\mathrm{aq}\right) $$

### Oxidation of nitric oxide by potassium permanganate (KMnO_4_)

It can be seen from Fig. 5 that the KMnO_4_ scrubbing mixture managed to convert about 45% of NO for the duration of the experimental run. The pH of the mixture reduced from 10.4 to 8.4 during the run, but this did not affect the oxidative power of the liquid solution much, as seen by the ORP values which hovered around 500–600 mV for the entire reaction. The drop in pH was probably due to the acidic nature of NO_2_ when absorbed and the low buffering capacity of the KMnO_4_ mixture. Unlike the chlorine-based oxidation agents, KMnO_4_ did not seem as sensitive to pH change as both its ORP values and NO conversion remained relatively stable throughout the reaction. The reaction mechanism for the conversion of NO to NO_2_ by KMnO_4_ is likely to have been the following (Chu et al. 2001; Fang et al. 2013):

19 $$ 3\mathrm{N}\mathrm{O}+2\mathrm{Mn}{\mathrm{O}}_4^{-}+{\mathrm{H}}_2\mathrm{O}\to 3\mathrm{N}{\mathrm{O}}_2+2\mathrm{Mn}{\mathrm{O}}_2\left(\mathrm{s}\right)+2\mathrm{O}{\mathrm{H}}^{-} $$

20 $$ \mathrm{N}\mathrm{O}+\mathrm{Mn}{\mathrm{O}}_4^{-}\to \mathrm{N}{O}_3^{-}+\mathrm{Mn}{\mathrm{O}}_2\left(\mathrm{s}\right) $$

21 $$ 3\mathrm{N}\mathrm{O}+\mathrm{Mn}{\mathrm{O}}_4^{-}+{\mathrm{H}}_2\mathrm{O}\to 3\mathrm{N}{O}_2^{-}+\mathrm{Mn}{\mathrm{O}}_2\left(\mathrm{s}\right)+2{\mathrm{H}}^{+} $$

As can be seen, all reactions resulted in the formation of MnO_2_, which is a solid precipitate. In all the experiments involving KMnO_4_ conducted here, a dark brown precipite was observed from early on in the experiments. This dark brown MnO_2_ precipitate was present everywhere in the setup and was etched in the tubings, the walls of the wet scrubber, and within the crevices of the spray nozzle. Cleaning was very challenging as the apparatus had to be dismantled and soaked in concentrated acid solution.

### Overall NO_x_ removal

**Fig. 8:**
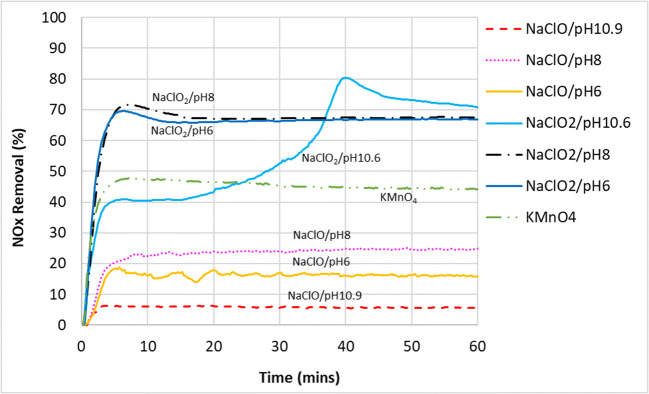
NO_x_ (NO+NO_2_) removal from the simulated exhaust gas using various scrubbing liquids

The removal of overall NO_x_, which is made up of NO and NO_2_, is shown in Fig. 8. Seawater, NaOH, and H_2_O_2_ were omitted from the graphs here as these chemical compounds were ineffective in NO_x_ removal. For the overall removal of NO_x_, the effectiveness of various oxidants or chemical compounds used under the present experimental conditions can be ranked as follows, from least to most effective:

Seawater, NaOH, H_2_O_2_ < NaClO < KMnO_4_ < NaClO_2_

It can be seen that the NO_x_ removal by NaClO was very low at pH 10.9, peaked when pH was lowered to 8, but then reduced slightly when pH was further lowered to 6. These NO_x_ removal figures were much lower compared to the oxidation rates of NO to NO_2_ that it achieved, suggesting that a significant amount of NO which were oxidized to NO_2_ could not be absorbed in the wet scrubber. As for the chlorites, both NaClO_2_/pH8 and NaClO_2_/pH6 exhibited very similar performances, managing around 65–70% of NO_x_ removal for the duration of their experiments. The fluctuation seen for NaClO_2_/pH10.6 aqueous mixture was likely to do with the lowering of the pH of the liquid mixture as the reaction progressed, leading to an increase in its oxidative properties.

**Fig. 9:**
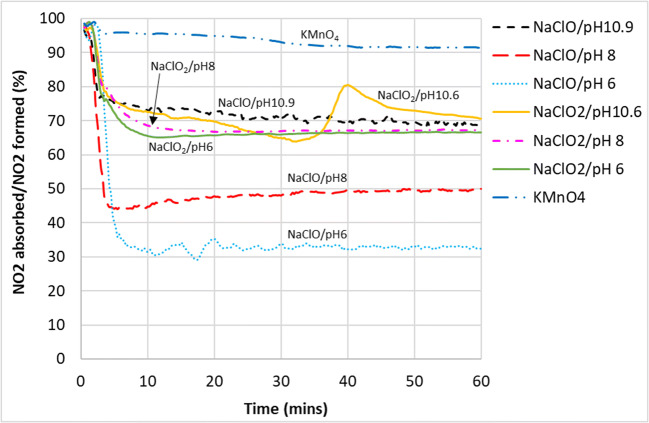
Ratio of NO_2_ absorbed over the total NO_2_ that was formed (converted to %) versus reaction time

**Fig. 10:**
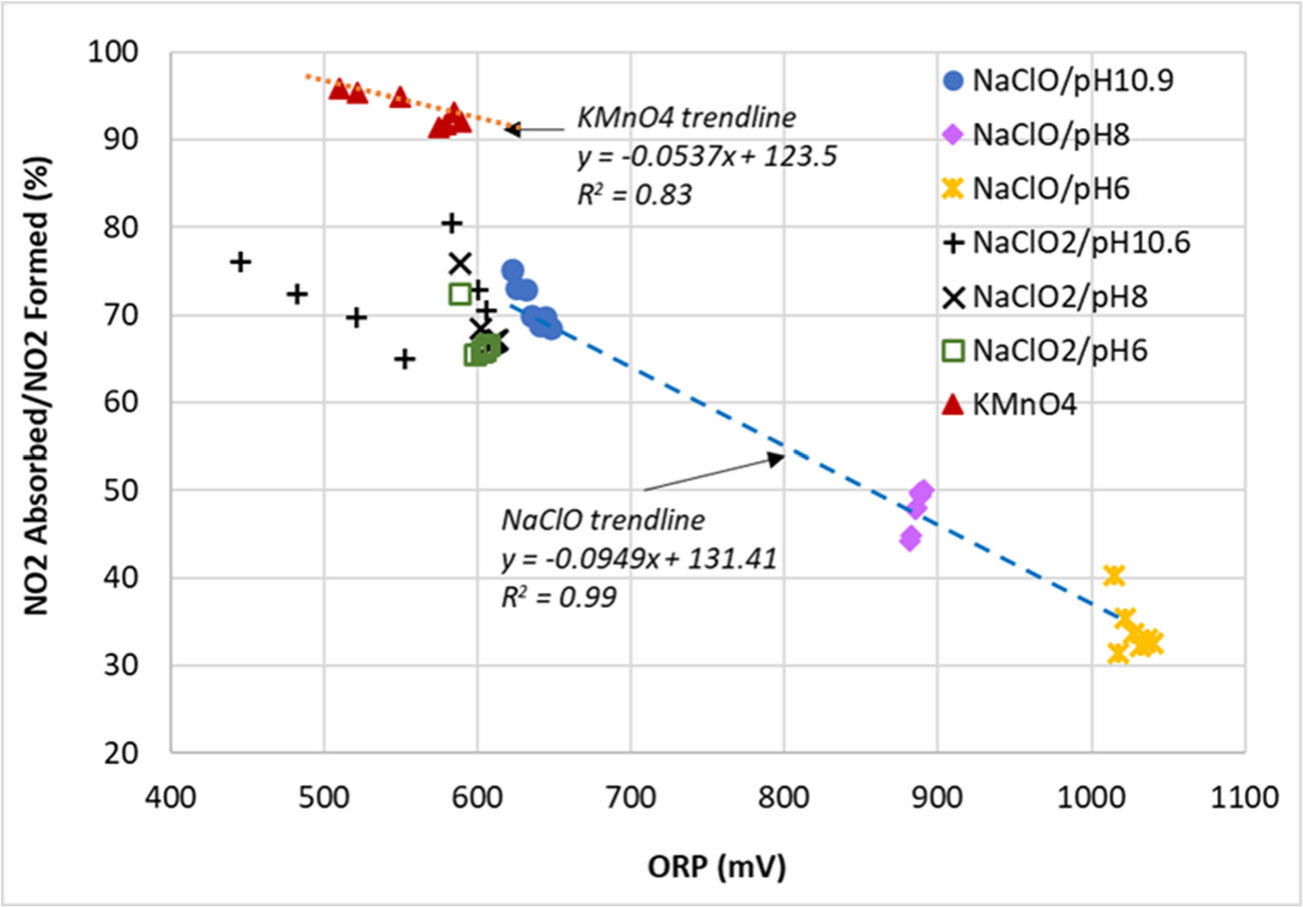
Ratio of NO_2_ absorbed over the total NO_2_ that was formed (converted to %) versus the oxidation potential

In order to get a better view of the NO_2_ absorption ability, Figs. 9 and 10 are plotted to show the ratio of NO_2_ absorbed over the amount of NO that was oxidized to NO_2_ for the duration of the experiment.

### Absorption and oxidation potential

From Fig. 10, a clear inverse relationship between NO_2_ absorption and oxidation potential of the various chemical compounds can be seen. NO_2_ absorption by NaClO showed the strongest inverse linear correlation with oxidation potential, with the linear regression *R*^2^ value at 0.99. However, it should not be expected that all the different points belonging to varous compounds fit nicely into one linear trendline as different chemical compounds have different reaction pathways even tough they all broadly followed the linear trendline.

The observations here is consistent with the work by Chang, Xi, and Zhang who used compounds which have very low oxidation potential such as Na_2_SO_3_, NaS, Na_2_S_2_O_8_, and NaHSO_3_ to improve the absorption of NO_2_ (Chang et al. 2004; Xi et al. 2020; Zhang et al. 2020). Chang and Xi further showed that in a low oxidation potential environment, NO_2_ can even be directly reduced to N_2_ gas, thereby avoiding the formation of nitrogen anions altogether (Chang et al. 2004; Xi et al. 2020).

It should also be pointed out here that these observations were in contradiction with the other school of thought which focused on increasing the oxidation potential in order to form high valency intermediates such as N_2_O_3_, N_2_O_4_, and N_2_O_5_ as these have higher solubility in aqueous solution (Brogren et al. 1998; Lin et al. 2016; Shao et al. 2019; Sun et al. 2015). The formation of these intermediates, especially N_2_O_5_, is heavily dependent on the residence time (Lin et al. 2016; Shao et al. 2019) and it was possible that the setup in this study could not provide sufficient residence time for these reactions to take place.

### Absorption by NaClO

As shown in Figs. 9 and 10, NaClO’s capacity to absorb NO_2_ increased with decreasing oxidation potential values in the pH range of 6–11. If NaClO is to be the oxidant of choice, a balance would have to be struck between achieving high NO oxidation rate, favored by high oxidation potential, versus absorbing the NO_2_ that will be formed, favored by a low oxidation potential. Compared with other oxidants, NaClO performed the poorest in terms of absorbing the NO_2_ that it formed from the oxidation of NO. However, NaClO could be ideal if the process requires only oxidation but not absorption.

### Absorption by NaClO_2_

In general, NaClO_2_ of various pH were more effective in absorbing the NO_2_ compared to NaClO due to its lower oxidation potential, managing to remove between 65 and 80% of NO_2_ that was formed during the reaction. In addition, it could also be that NaClO_2_ has additional reaction pathways for the absorption of NO_2_ in the aqueous phase on top of the reactions shown in Eqs. 8 and 9 (Brogren et al. 1997):

22 $$ 4\mathrm{N}{\mathrm{O}}_2+\mathrm{C}\mathrm{l}{\mathrm{O}}_2^{-}+4{\mathrm{O}\mathrm{H}}^{-}\to 2\mathrm{N}{\mathrm{O}}_3^{-}+\mathrm{C}{\mathrm{l}}^{-}+2{\mathrm{H}}_2\mathrm{O} $$

23 $$ 4\mathrm{N}{\mathrm{O}}_2+\mathrm{C}\mathrm{l}{\mathrm{O}}_2^{-}+2{\mathrm{O}\mathrm{H}}^{-}\to 2\mathrm{N}{\mathrm{O}}_3^{-}+\mathrm{C}{\mathrm{lO}}^{-}+{\mathrm{H}}_2\mathrm{O} $$

### Absorption by KMnO_4_

It can be seen from Fig. 9 that KMnO_4_ was the most effective in the absorption of NO_2_—almost all the NO that was oxidized to NO_2_ was subsequently absorbed into the aqueous phase. One possiblity was that KMnO_4_ oxidized NO directly to nitrate ions in the aqueous phase as shown in Eqs. 20 and 21 instead of the other gaseous intermediates such as NO_2_. Nevertheless, KMnO_4_ still trailed chlorite ions in terms of overall NO_x_ removal and the staining of pipes, pumps, and nozzles among the wet scrubbing equipment poses a significant problem for it to be considered the oxidant of choice. Brogren reported that the formation of the MnO_2_ precipitate can be avoided under very high alkaline conditions—when the solution contains more than 3 mol/L of hydroxide ions, $\mathrm{Mn}{\mathrm{O}}_4^{-}$ will be formed instead of MnO_2_ (Brogren et al. 1997) . However, it was also reported in the same study that NO_x_ removal will be suppressed under such high pH conditions. Furthermore, it will also be quite costly to maintain such a high pH in a large-scale operation.

### Absorption of nitrogen dioxide﻿ (NO_2_)

**Fig. 11:**
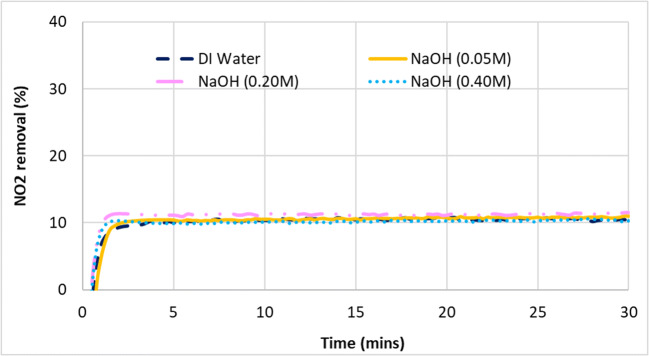
NO_2_ gas removal from the simulated exhaust gas using DI water versus scrubbing mixtures of increasing alkalinity (NaOH 0.05–0.40 M)

In this additional study, selected chemical compounds were reacted with NO_2_ in the wet scrubber in order to gain a clearer understanding of its absorption and removal in the gas–liquid reaction. From Fig. 11, it can be seen that deionized (DI) water could only remove around 10% of the NO_2_ in the exhaust gas. Addition of NaOH up to 0.40 M to increase the alkalinity of the scrubbing liquid did not improve the absorption of NO_2_ at all. The results seen here are in contrast with some of the previous reported literature which suggested significant levels of NO_2_ absorption in the aqueous phase is possible after the oxidation of NO to NO_2_ has been achieved (Brogren et al. 1998; Kurpoka 2011). In one such example, Brogren reported that around 50–60% of NO_2_ was successfully removed by NaOH between pH 9 and 12 (Brogren et al. 1998). However, it was consistent in the study by Chang et al. which showed NaOH as high as pH 13 had no effect on absorbing NO_2_ (Chang et al. 2004).

**Fig. 12:**
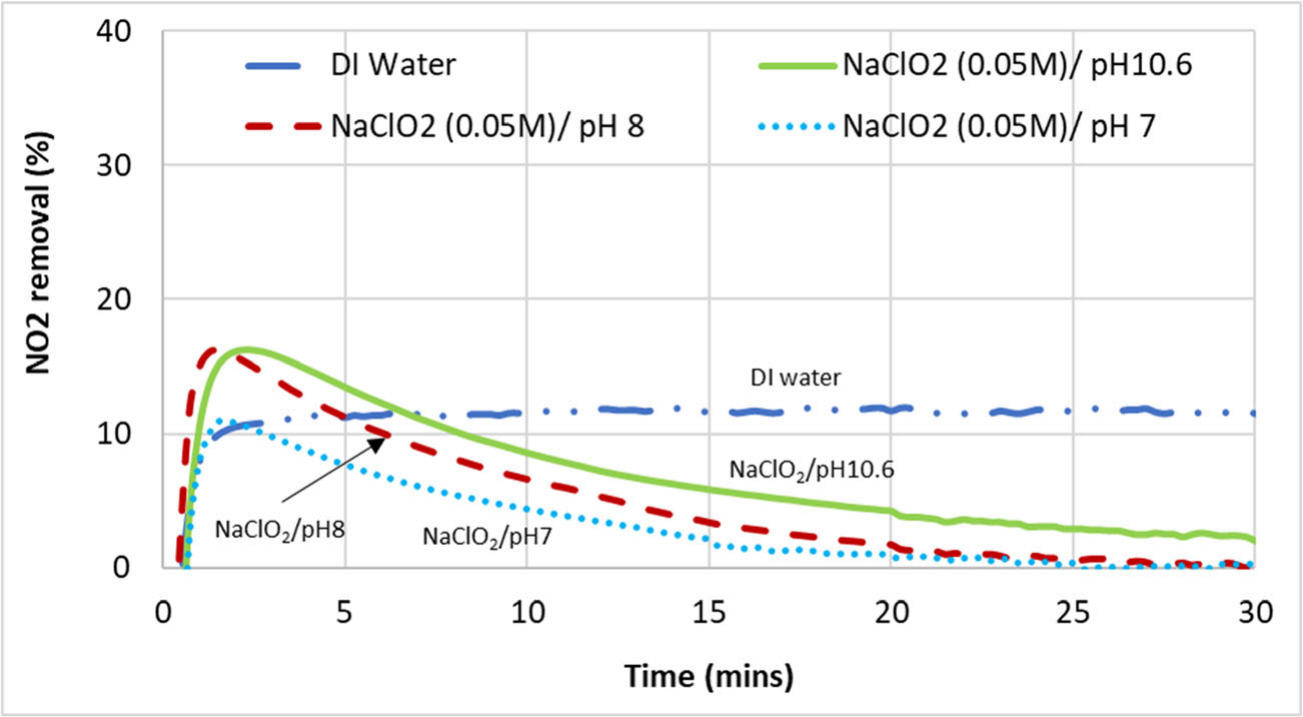
NO_2_ gas removal from the simulated exhaust gas using DI water versus scrubbing liquids of increasing oxidation potential (NaClO_2_/pH10.9 < NaClO_2_/pH8 < NaClO_2_/pH7)

Brogren also reported that the addition of sodium chlorite under alkaline conditions increased the absorption of NO_2_, up to almost 80% removed when 0.6 M was used. However, it can be seen from Fig. 12 that the addition of NaClO_2_ in the scrubbing mixture decreased the amount of NO_2_ absorbed. Since Fig. 11 already shows that NO_2_ absorption was not directly dependent on pH, it follows that the diminishing capacity to absorb NO_2_ seen in Fig. 12 when the pH of NaClO_2_ was lowered from 10.6 to 6 likely has less to do with the increasing acidity of the aqueous solution but rather due to the increasing oxidation potential.

Although NO_2_ gas is at least five times more soluble in the aqueous phase than NO (Table 3), its solubility was clearly still insufficient for the significant absorption and removal of NO_x_ from the exhaust. Absorption and removal of NO_2_ required more than can be provided by an alkaline mixture such as NaOH. Increasing the oxidizing potential in attempt to form higher valency nitrogen intermediates which have higher solubility such as N_2_O_3_, N_2_O_4_, and N_2_O_5_ did not improve the absorption but made it worse, contrary to reported literature (Brogren et al. 1998; Lin et al. 2016; Shao et al. 2019; Sun et al. 2015). This was consistent with the findings discussed previously Section 3.2.2.1.

### Analysis of aqueous solution

**Fig. 13:**
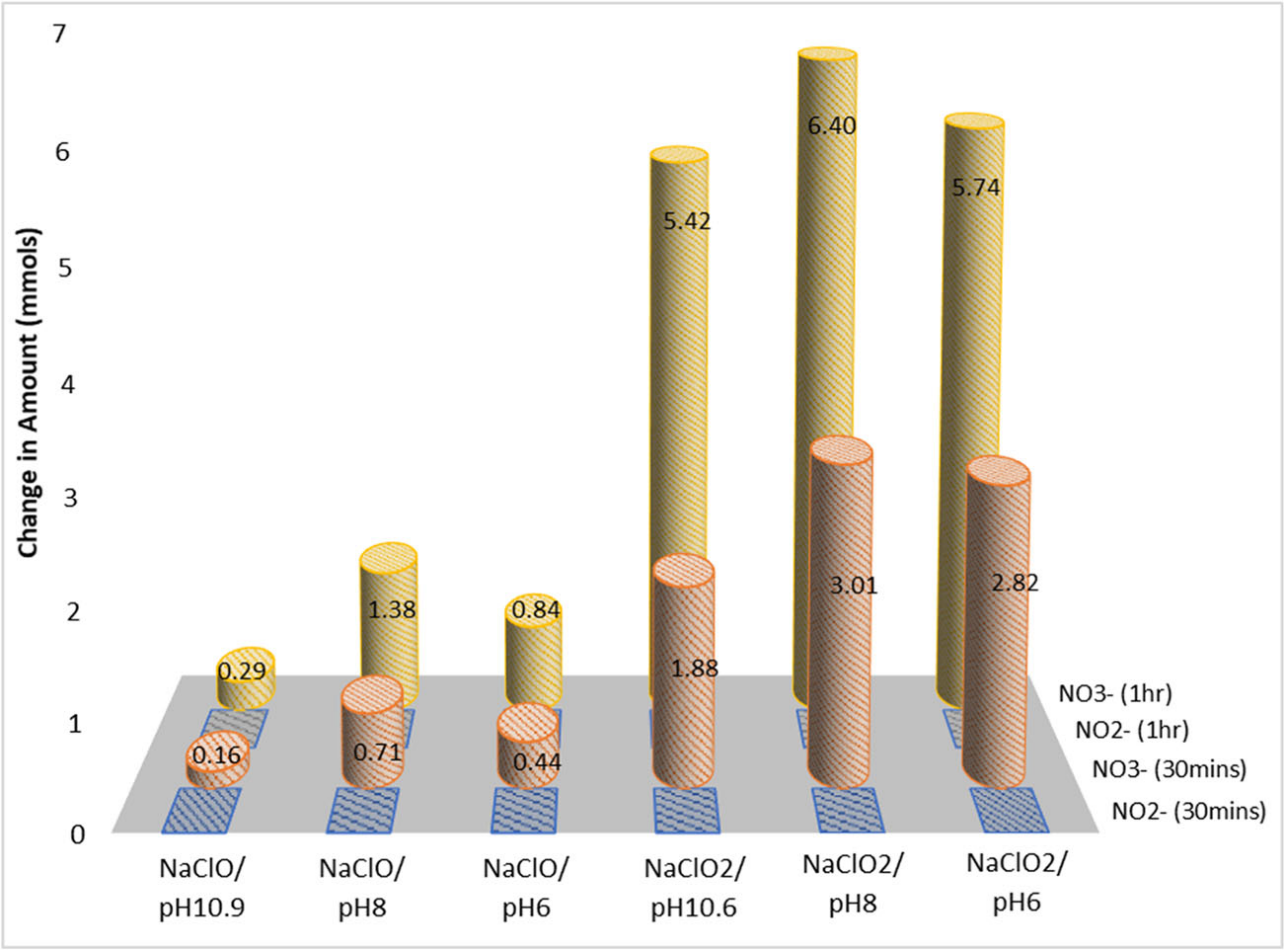
Amount of aqueous nitrogen of various reactions using NaClO and NaClO_2_ quantified by ion chromatography

The analysis of aqueous samples from scrubbing mixtures which could remove NO_x_ at least partially are presented here. NO and NO_2_ gases captured in the gas–liquid reaction in the wet scrubber should end up as either nitrites or nitrates in the aqueous phase. From Fig. 13, it can be seen that the nitrogen existing in the aqueous phase were all in nitrate form. This was because the residue oxidizing agent in the liquid phase will oxidize all the nitrites into nitrates. This is advantageous as nitrates are the more stable in the aqueous phase. However, one area of concern is if part of the scrubbing liquid needs to be treated and discharged into the ocean during voyage. According to existing IMO guidelines for wastewater discharge from vessels to the ocean, high levels of nitrates may cause algae bloom, especially near the coastal areas, and are hence subjected to an upper discharge limit, in contrast to sulfates and chlorides which are considered to be naturally occurring in seawater and can be discharged freely (IMO 2015).

In Table 5, the amount of nitrates in the aqueous system was compared to the calculated theoretical amount of NO_x_ that was removed based on the results from the flue gas analyzer. It can be seen that for NaClO samples of various starting pH, only around 35–50% of the NO_x_ captured showed up as nitrates in the aqueous system. This range was around 70–80% for NaClO_2_ samples.

**Table 5:** Amount of NO_x_ removed by the various scrubbing liquids and the amount eventually converted to nitrate ions in the aqueous phase

Scrubbing liquid	Total amount of nitrates formed (mmol)	Total amount of NO_x_ removed (mmol)	Percentage of removed NO_x_ converted to nitrates (%)
NaClO	0.29	0.82	35.4
NaClO/pH 8	1.38	2.90	47.6
NaClO/pH 6	0.84	1.99	42.2
NaClO_2_	5.42	7.06	76.8
NaClO_2_/pH 8	6.40	8.32	76.9
NaClO_2_/pH 6	5.74	8.23	69.7
KMnO_4_	–	5.65	–

The unaccounted nitrogen between the gaseous and aqueous phases could at least be partially attributed to the assumption that the system followed the ideal gas law when converting the gaseous concentration values from ppm(v) to mole. Second, it was also possible that the NO_2_ that was absorbed by the aqueous mixture was unstable and a portion of it could have desorbed from the scrubbing liquid before the quantitative analysis was carried out (within 24 h), on account of Eqs. 8 and 9 being reversible reactions. These unstable absorbed nitrogen includes non-anionic aqueous forms such as NO_2_ (aq) or HNO_2_ (aq) (Yang et al. 2016). Third, they could simply have been reduced to N_2_ gas especially when under low oxidation potential conditions (Chang et al. 2004).

These results clearly showed that in the operating pH range from 6 to 11, NaClO_2_ was more successful in not only oxidizing NO to NO_2_ (Fig. 5), but also in absorbing (Figs. 9 and 13) and retaining (Table 5) the NO_2_ in the aqeuous phase, compared to NaClO. It could effectively oxidize NO to NO_2_ without needing a high ORP environment in the bulk phase likely due to its ability to decompose to its more oxidative form, ClO_2_, at the gas–liquid interface, so the subsequent absorption of NO_2_ which required a lower ORP environment was not inhibited.

For both NaClO and NaClO_2_, the reaction at pH 8 registered the highest amount of nitrates in the aqueous system. The balance between the ability to oxidize NO to NO_2_ and absorb the NO_2_ formed was seen around this pH region. Deshwal speculated a similar concept of this balance when studying NaClO_2_ but without arriving at an optimal pH as the work carried out was in the acidic pH range (Deshwal et al. 2008). A study by Han et. al. on NO removal using NaClO_2_ between the pH of 2.4 and 8.0 also showed that the absorption of NO_2_ that was formed from the oxidation of NO was highest at pH 8.0 (Han et al. 2019).

### Consumption of reactants

**Fig. 14:**
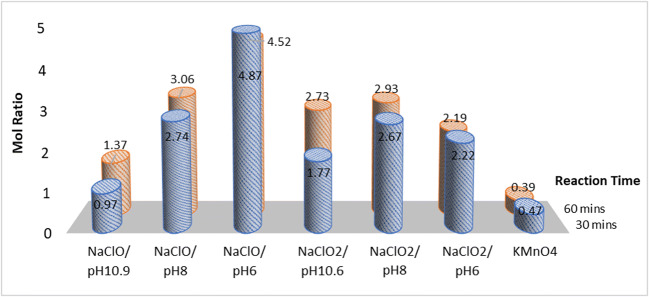
The mole ratio of the reactant consumed per mole of NO_x_ removed

The mole ratio of the reactant consumed over the amount of NO_x_ that was removed is shown in Fig. 14. It can be seen that for NaClO, the consumption of reactants for every mole of NO_x_ removed increased with decreasing pH, for the pH range of 6–11. This was because as the pH shifted from 11 to 6, the dominant form of the chlorine oxidants also shifted from ClO^−^ to HOCl. The latter, while having a stronger oxidation strength, was also more volatile, leading to significant losses to the scrubber exhaust and a high reactant consumption rate. If NaClO is the oxidant of choice, the operating pH should be in the region of 8, as this range provided a balance between effectiveness of NO_x_ removal versus reactant consumption rate. In this experimental setup, about 3 mol of ClO^−^ oxidant was consumed for every mole of NO_x_ removed at this operating pH.

Except for NaClO/pH10.9 and NaClO_2_/pH10.6, all reactions showed similar reactant consumption rates at the midpoint and end of reaction, suggesting that the consumption rates of reactants were linear throughout the experimental duration. These two liquid mixtures saw increasing reactant consumption because their pH started high but gradually dropped throughout the reaction, leading to higher reactant losses to the gaseous phase.

The various samples of NaClO_2_ achieved between 2 and 3 moles of reactant consumed for every mole of NO_x_ removed; this was a better performance compared to NaClO, which ranged between 3 and 5 mol (with the exception of NaClO/pH10.9 as the NO_x_ removal for that sample was quite insignificant). Of all the reactants studied here, KMnO_4_ had the lowest reactant consumption rate, achieving around half a mole of reactant consumed for every mole of NO_x_ removed. Unlike chlorine-based oxidants which tended to partition into more volatile forms especially at lower pH, the permanganate oxidant tends to precipitate out of the aqueous phase as solid deposits instead. It is able to achieve this low reactant consumption rate since it is not volatilized and lost via the exhaust like chlorine-based oxidants.

**Fig. 15:**
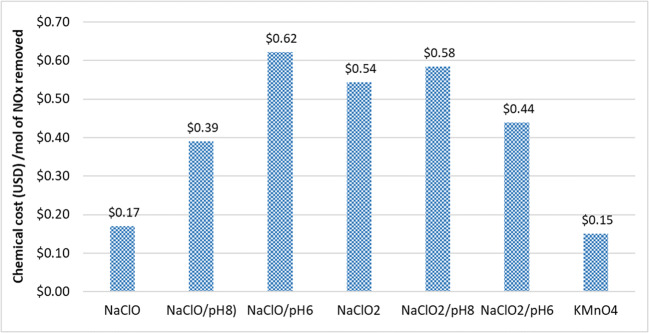
The estimated chemical cost of various reactant per mole of NO_x_ removed

Based on the amount of chemicals consumed in Fig. 14 and the cost of chemicals in Table 6, the estimated chemical cost per mole of NO_x_ removed was estimated in Fig. 15. It can be seen from Table 6 that the bulk cost of industrial chemicals from cheapest to most expensive is NaClO < NaClO_2_ < KMnO_4_. Although KMnO_4_ is the most expensive chemical, it is still the most cost-effective after accounting for its high utilization rate, at approximately USD 0.15 per mole of NO_x_ removed. Although NaClO was less efficient than NaClO_2_ in terms of reactant consumption, they did not differ significantly after accounting their cost—both lie around the range of USD 0.39–0.62 per mole of NO_x_ removed. Therefore, if these two compounds are being considered for usage in a system, the choice would likely be decided by other factors instead of costs.

**Table 6:** Cost estimation of various scrubbing liquid systems used

No	Scrubbing liquid	Reactant cost (per mole)	Cost of HCl needed for pH adjustment (per mole)	Total cost (reactant + acid) (per mole)
1	NaClO	USD 0.124	USD 0.000	USD 0.124
2	NaClO/pH8	USD 0.124	USD 0.003	USD 0.127
3	NaClO/pH6	USD 0.124	USD 0.013	USD 0.137
4	NaClO_2_	USD 0.199	USD 0.000	USD 0.199
5	NaClO_2_/pH8	USD 0.199	USD 0.001	USD 0.200
6	NaClO_2_/pH6	USD 0.199	USD 0.002	USD 0.201
7	KMnO_4_	USD 0.392	USD 0.000	USD 0.392

Bulk cost of chemicals was estimated from industrial chemical aggregator sites, namely, chembid.com, alibaba.com, and diytrade.com

### Scalability

**Fig. 16:**
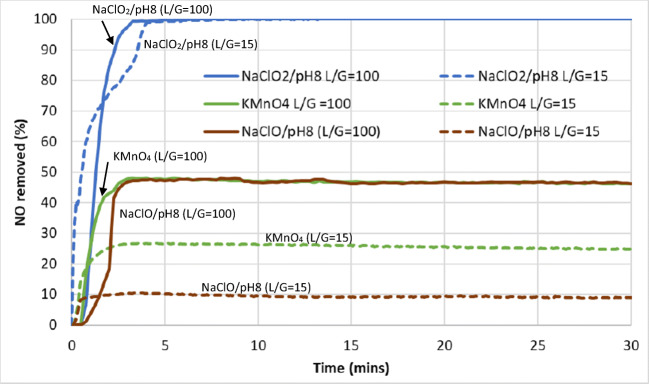
Nitric oxide (NO) removal from the simulated exhaust gas with various scrubbing liquids at L/G ratios of 100 L/m^3^ vs. 15 L/m^3^, respectively

**Fig. 17:**
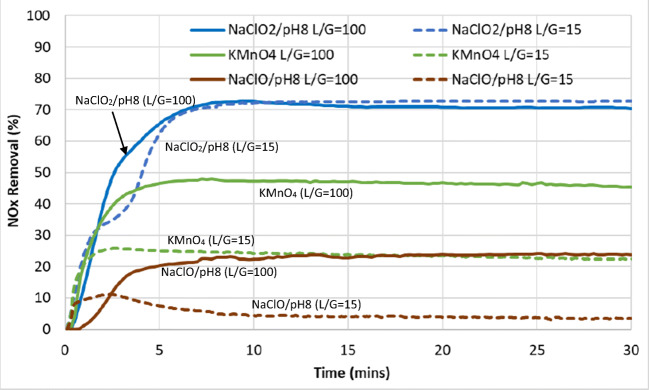
NO_x_ (NO+NO_2_) removal from the simulated exhaust gas using various scrubbing liquids

The optimal conditions for the best three scrubbing compounds, namely, NaClO_2_, KMnO_4_, and NaClO, were selected for further experimental runs at a significantly higher flowrate which are closer to industrial wet scrubbers. This was carried out to observe the behavior of each of these compounds when scaled to a liquid-to-gas ratio that is closer to industrial norms. Focus was placed on NO removal as it is the more challenging pollutant compared to SO_2_. The L/G ratios were decreased from 100 to 15 L/m^3^ and the results are shown in Figs. 16 and 17.

It can be seen that the conversion of NO and removal of NO_x_ by NaClO_2_ were very similar for both L/G ratios. However, this was not so for KMnO_4_ and NaClO, which saw a reduction in capacity for the oxidation of NO and removal of NO_x_ when the L/G ratio was reduced to 15. KMnO_4_ encountered a reduction of 40% in its capacity to oxidize NO to NO_2_ and in the removal of NO_x_. As for NaClO, this reduction of capacity was around 80% for both the oxidation of NO and removal of NO_x_.

This kinetic limitation in the capacity of KMnO_4_ and NaClO to oxidize NO and absorb NO_x_ when the L/G ratio was reduced (i.e., gas flowrate was increased and liquid flowrate reduced) showed that these two compounds were more limited in their mass transfer capacities compared to NaClO_2_. As NaClO_2_ showed a maximum conversion (100%) of NO to NO_2_ for both high and low L/G ratios studied here, it is likely that it has the mass transfer capacity to remain effective at even lower L/G ratios. Therefore, NaClO_2_ would likely be the most effective compound compared to KMnO_4_ and NaClO when scaling up to industrial size requires much higher gas flowrates compared to liquid flowrates. The latter two compounds may still be used if a significant increase in concentration can improve their effectiveness, but further work is needed to verify this at low L/G ratios.

## Conclusions

For the reaction with SO_2_, full removal of SO_2_ proceeded quite readily and was achieved by nearly all the different types of scrubbing mixtures that were tested. This is because SO_2_ gas is very soluble in the aqueous phase. The absorption of SO_2_ in the aqueous phase by the various gas–liquid reactions were likely influenced by three factors, namely, pH, the ionic concentration in the scrubbing mixture in terms of both its overall ionic strength and concentration of sulfate ions, and oxidation potential. An effective scrubbing liquid for the effective removal of SO_2_ gas should have high pH or alkalinity, low in ionic strength and sulfate ions, and oxidative in nature.

As for NO_x_ removal, the effectiveness of various chemical compounds used can be ranked as follows, from least to most effective: seawater, NaOH, H_2_O_2_ < NaClO < KMnO_4_ < NaClO_2_. The first three, seawater, NaOH, and H_2_O_2_, had little or no effect. NaClO was somewhat effective when the pH was lowered to 9 and below, when the hypochlorite ions shifted to its oxidative form, HOCl. Following that was KMnO_4_ which was moderately effective, while NaClO_2_ was the most effective, especially when the pH was below 10. When the L/G ratio was reduced from 100 to 15 L/m^3^, NaClO_2_ showed no changed in its effectiveness for NO_x_ removal while NaClO and KMnO_4_ showed a reduction in 80 and 40%, respectively. This showed that NaClO_2_ is the most reactive and suitable for scaling up to industrial size (higher gas flowrate, lower liquid flowrate conditions) while NaClO and KMnO_4_ would probably require higher concentrations to make up for their kinetic and mass transfer limitations.

For achieving high NO_x_ removal, the scrubbing liquid mixture must be effective in (1) oxidizing NO to NO_2_, (2) absorbing the NO_2_ into the aqueous phase, and (3) retaining the nitrogen in the aqueous phase as anions. Seawater, NaOH, and H_2_O_2_ were ineffective in NO_x_ removal because they had difficulty oxidizing NO to NO_2_. NaClO was effective in oxidizing NO to NO_2_ after it partitioned into its HOCl form when the pH was reduced below 9. However, it was not very effective in absorbing and retaining NO_2_ in the aqueous phase, especially when the pH was lowered to below 9—up to half of the NO_2_ that was absorbed likely desorbed back into the atmosphere after a short period of time.

Successful oxidation of NO to NO_2_ did not necessarily translate to high NO_x_ removal as the absorption of NO_2_ proved to be a challenge although it is approximately five times more soluble than NO in the aqueous phase. Alkalinity was not a factor in the absorption of NO_2_ into the aqueous phase as increasing the NaOH concentration had no effect on it. Rather, NO_2_ absorption showed an inverse relationship with oxidation potential in this study. The seeming relationship between NO_2_ absorption and pH was likely coincidental since the oxidation potential of chlorine-based oxidants are also pH dependent.

NaClO_2_ was superior compared to NaClO in all three categories of oxidizing, absorption, and retention of NO in the pH range of 6–11. It could effectively oxidize NO to NO_2_ without needing a high ORP environment in the bulk phase likely due to its ability to decompose to its more oxidative form, ClO_2_, at the gas–liquid interface, so the subsequent absorption of NO_2_ which required a lower ORP environment was not inhibited.

In the pH range of 6–11 studied here, the pH at around the region of 8 provided an optimal balance between oxidation versus both absorption/retention and reactant utilization for NaClO and NaClO_2_, respectively. Operating at an optimal pH was important as to minimize reactant losses to the atmosphere as both NaClO and NaClO partitioned into a gaseous state at lower pH.

Although KMnO_4_ was less effective than NaClO_2_ in terms of overall NO_x_ removal, it was very effective in absorbing and retaining the NO_2_ in the aqueous phase. In fact, it was possible that this seemingly high NO_2_ absorption could be because KMnO_4_ was able to oxidize NO into the aqueous phase without forming gaseous intermediates such as NO_2_. KMnO_4_ also had the lowest reaction consumption rate, with only half a mole utilized for every mole of NO_x_ removed, compared to 2–3 mol of NaClO_2_ or 3–5 mol of NaClO needed for every mole of NO_x_ removed. This was because unlike the chlorine-based oxidants, KMnO_4_ does not partition into a more volatile form, leading to less reactant losses to the atmosphere. However, KMnO_4_ has a tendency to precipitate in the form of MnO_2_ which caused clogging and was very difficult to remove.

In terms of chemical cost per mole of NO_x_ removed, KMnO_4_ is the most cost effective which NaClO and NaClO_2_ were similar in range. As each of the chemical reactant compared here have their own advantages and disadvantages, the choice of the most suitable reactant will still depend on the actual design of the wet scrubbing system. Nevertheless, this comparison exercise enabled a deeper understanding of the reaction mechanisms and behavior of the reactants during reaction.

## Data Availability

The datasets used or analyzed during the current study are available from the corresponding author on reasonable request.
